# Spatial Distribution of Prostate Cancer Missed by MRI‐Targeted Biopsy, Effect of a Perilesional Template, and Restaging in a Radical Prostatectomy Cohort

**DOI:** 10.1002/pros.70201

**Published:** 2026-05-26

**Authors:** Ruth Himmelsbach, Julia Franz, Cordula Jilg, Christian Gratzke, August Sigle

**Affiliations:** ^1^ Department of Urology Faculty of Medicine, University of Freiburg ‐ Medical Centre Freiburg Germany

**Keywords:** fusion biopsy, image‐guided biopsy [MeSH], prostatic neoplasms [MeSH]

## Abstract

**Purpose:**

Systematic biopsy (SB) remains recommended in addition to MRI‐targeted biopsy (TB) because TB alone may miss clinically significant prostate cancer (sPC). We evaluated the spatial distribution of sPC and clinically insignificant prostate cancer (iPC) detected by SB in relation to MRI lesions and assessed the effect of perilesional template (PLT) in terms of cancer detection.

**Materials and Methods:**

We retrospectively analyzed 1043 men who underwent combined transperineal MRI/TRUS fusion biopsy. Spatial distribution between MRI lesions and cancer‐positive SB cores was calculated. Hypothetical PLTs with 5, 10, and 15 mm were modeled. Radical prostatectomy (RP) whole‐mount histopathology served as the reference standard.

**Results:**

sPC was detected in 521/1043 (50.0%) men. SB alone identified sPC in 98 patients. The mean distance to the nearest sPC core was significantly shorter than for iPC (7.8 vs. 12.4 mm; *p* < 0.001). Cumulative sPC detection increased up to approximately 10 mm from the lesion border and plateaued thereafter, whereas iPC detection increased linearly beyond 10 mm. A 10mm‐PLT would have detected 94.3% of all cancers and avoided 3.7% of iPC diagnoses, while missing 2.5% of sPC. Extending the margin to 15 mm did not meaningfully improve sPC detection but increased iPC detection. Use of 10mm‐PLT would have resulted in 10.5% upgrades to sPC in RP compared to 5.9% in the TB + SB cohort.

**Conclusions:**

sPC is spatially concentrated near MRI lesions, whereas iPC is more widely distributed. A 10mm‐PLT captures most sPC and provides an empirical basis for the guideline‐recommended perilesional margin. Prospective validation is required before SB can be safely replaced.

## 1 Introduction

Current European Association of Urology guidelines recommend a magnetic resonance imaging (MRI) based diagnostic pathway for prostate cancer (PC). In biopsy‐naïve patients with a suspicious MRI finding, typically defined as PI‐RADS ≥ 3 depending on the clinical context, a combined approach of targeted biopsy (TB) and systematic biopsy (SB) is recommended. Systematic biopsy traditionally consists of up to 12 cores obtained from predefined sectors of the prostate. This recommendation is based on evidence demonstrating that the combination of SB and TB increases the detection rate of significant prostate cancer (sPC) by approximately 5.2–11% compared with TB alone [1, 2].

More recently, several studies have identified targeting errors, defined as the failure to accurately sample an MRI‐visible lesion, as a major contributor to the limited sensitivity of MRI‐TB alone [3, 4]. Other factors, including MRI‐invisible lesions, radiological misinterpretation, and intralesional histological heterogeneity, have also been described; however, their contribution to missed sPC appears to be comparatively lower [5, 6].

Building on this insight, Brisbane et al. demonstrated in a retrospective analysis that approximately 90% of sPC was located within the MRI‐visible lesion or within a 10 mm perilesional region [7]. These findings further support the concept that imprecision in lesion targeting represents a major reason for missing sPC when SB is omitted.

Against this background, we hypothesized that the spatial distribution of iPC and sPC detected by SB differs in relation to MRI lesions with negative biopsy findings. Furthermore, we aimed to evaluate whether a perilesional template (PLT) could achieve a detection rate of sPC comparable to the current standard of combined biopsy while potentially reducing procedural invasiveness and the detection of clinically insignificant disease. Validation of PLT‐based biopsy strategies was performed using whole‐mount histopathological analysis of radical prostatectomy (RP) specimens, which served as a reference standard.

## 2 Patients and Methods

### 2.1 Study Population

Between October 2015 and May 2020, 1087 men underwent combined transperineal MRI/TRUS fusion prostate biopsy (pFBx) at the Department of Urology at the University Hospital Freiburg. Forty‐five patients were excluded due to incomplete clinical data. A total of 1043 patients were included in the final analysis.

To evaluate diagnostic accuracy, the RP cohort with whole‐mount histopathological analysis of surgical specimens served as the reference standard.

Upgrading was defined as an increase in International Society of Urological Pathology (ISUP) grade group between pFBx and whole‐mount histopathology.

### 2.2 Definition of Terms

Clinically, iPC was defined as ISUP grade group 1 (Gleason score 3 + 3 = 6). Clinically, sPC was defined as ISUP grade group ≥ 2.

### 2.3 Biopsy Procedure

All robot‐assisted pFBx procedures were performed using the same biopsy platform (iSRobot Mona Lisa™®, Biobot Surgical, Singapore). The procedure was performed as a combined strategy consisting of TB of MRI‐suspicious lesions followed by SB within the same session. Procedural details have been described previously [8]. Systematic sampling was performed according to the Ginsburg scheme [9]. The number of cores per sector depended on prostate volume. All procedures were performed in the lithotomy position under general anesthesia. Antibiotic prophylaxis was not administered routinely.

### 2.4 Spatial Analysis and Definition of Targeting Error

Within the total biopsy cohort, we identified men with discordant results between TB and SB. Discordant results were defined as the absence of cancer in TB with detection of iPC in SB, or upgrading to sPC based on SB findings. For spatial analysis, the prostate was divided into sextants. A true targeting error was assumed when an SB core positive for cancer was located within the same sextant as a cancer‐negative MRI‐targeted lesion. Detection in an adjacent sextant was considered potentially attributable to targeting error. Detection outside the same or adjacent sextant was considered unlikely to be due to targeting error.

**Figure 1:**
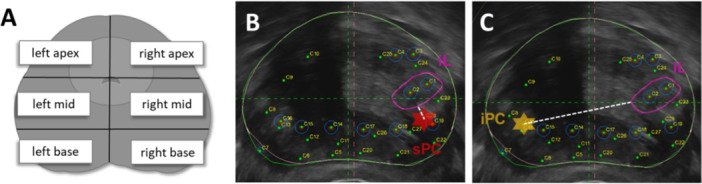
(A) division of prostate in sextants; (B) distance of cancer‐free MRI index lesion to the closest sPC core; (C) distance of cancer‐free MRI index lesion to the closest iPC core. iL, index lesion; sPC, iPC, insignificant prostate cancer; significant prostate cancer. [Color figure can be viewed at wileyonlinelibrary.com]

In case of discordant biopsy results, the exact distance from the MRI lesion border to the closest iPC core and sPC core was determined using the biopsy plan provided by the biopsy platform (Figure 1).

**Figure 2:**
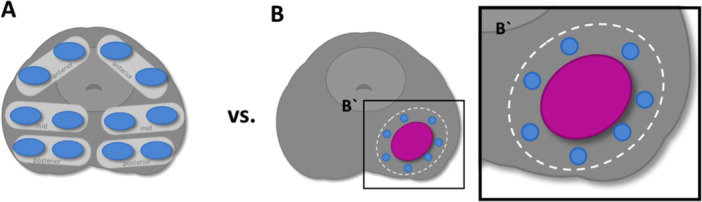
(A) Standard systematic biopsy in accordance with the Ginsburg scheme; (B) perilesional template. [Color figure can be viewed at wileyonlinelibrary.com]

To model the hypothetical effect of a PLT, a circular perilesional margin defined by a specified radial distance from the lesion border was applied retrospectively. Only biopsy cores within this area were considered for detection rate calculations. A schematic illustration of the PLT is shown in Figure 2.

### 2.5 Data Collection and Statistical Analysis

Demographic and clinical data were extracted by reviewing patients' electronic medical records. Baseline characteristics included age, previous biopsy, active surveillance, PSA, prostate volume (MRI or TRUS), MRI findings according to PI‐RADS, lesion localization, index lesion volume, number of lesions, and histopathological findings according to the ISUP classification.

Continuous variables are reported as medians with interquartile ranges (IQRs) or means with standard deviations (SDs). Categorical variables are presented as absolute numbers and percentages. Group comparisons were performed using chi‐squared tests, with post hoc analyses and Bonferroni correction where applicable.

A *p*‐value < 0.05 was considered statistically significant. All statistical analyses were conducted using SPSS© Statistics version 29 (IBM Corp., Armonk, NY, USA). Artificial intelligence (AI)–based language editing (ChatGPT, OpenAI, USA) was used solely for linguistic revision and improvement of grammar and style. No scientific content, data analysis, or interpretation was generated by AI.

## 3 Results

### 3.1 Baseline Characteristics and Cancer Detection Rates

Detailed patient characteristics of the overall cohort and the RP cohort are shown in Table 1. A total of 1043 men with combined pFBx were included. The median age was 67.0 years (IQR 61.0–72.0). Median PSA and prostate volume were 8.8 ng/mL (6.0–12.6) and 53.0 mL (38.5–75.0), respectively. Overall, 36.5% (381/1043) of men had undergone at least one prostate biopsy before, and 13.5% were under active surveillance. The most frequent PI‐RADS score of the index lesion was 4 (545/1043; 50.3%). The index lesions were classified as PI‐RADS 3 in 175/1043 patients (16.1%) and PI‐RADS 5 in 238/1043 patients (22.0%). The median index lesion volume was 0.58 ml (0.32–‐1.14). More than half of the patients had more than one suspicious lesion on MRI. Overall, 649/1043 (62.2%) men were diagnosed with PC. Combined biopsy detected sPC in 521/1043 (50.0%) patients. Of these, 38/521 (7.3%) were diagnosed by TB alone and 98/521 (18.8%) by SB alone. In 98/1043 patients, SB led to an upgrade to sPC. Without SB, 59 ISUP 2, 24 ISUP 3, 14 ISUP 4, and 1 ISUP 5 PCs would have been missed. Overall, 128/1043 (12.3%) patients were diagnosed with iPC. In 72/1043 (6.9%) cases, iPC was solely diagnosed by SB. Of 649 men diagnosed with PC, 239 underwent RP in our institution after detailed discussion of potential treatment options.

**Table 1:** Baseline characteristics.

Characteristic	All men	RP cohort
Cases, *n*	1043	239 (22.91%)
Age (years), median, IQR	67.0 (61.0–72.0)	67 (62.0–72.0)
Previous biopsy, *n* (%)	381 (36.5%)	76 (31.8%)
Active surveillance, *n* (%)	141 (13.5%)	30 (12.6%)
PSA (ng/mL), median, IQR	8.8 (6.0–12.6)	9.05 (6.1–14.0)
Volume (mL), median, IQR	53.0 (38.5–75.0)	47.0 (36.9–63.5)
PI‐RADS, *n* (%)		
*n*/a	80 (7.4%)	10 (4.2%)
1	1 (0.1%)	0 (0.0%)
2	45 (4.2%)	6 (2.5%)
3	175 (16.1%)	21 (8.8%)
4	545 (50.3%)	112 (46.9%)
5	238 (22.0%)	90 (37.7)
Target localization, *n* (%)		
Unilateral	595 (57.0%)	130 (54.4%)
Bilateral	448 (43.0%)	109 (45.6%)
Non‐peripheral zone	221 (21.2%)	113 (47.3%)
Peripheral zone	444 (42.6%)	38 (15.9%)
Bizonal	378 (36.2%)	88 (36.8%)
Index lesion volume (mL), median, IQR	0.58 (0.32–1.14)	0.6 (0.3–1.4)
Number of lesions, *n* (%)		
1	481 (46.1%)	116 (48.5%)
2	394 (37.8%)	87 (36.4%)
3	136 (13.0%)	28 (11.7%)
4 or more	32 (3.1%)	8 (3.3%)
Number of cores, median, IQR		
Total	35 (31–40)	35 (31–39)
From target	5 (3–7)	4 (4–7)
Systematic	31 (26–34)	30 (26–32)
Cancer grading according to ISUP, *n* (%)		
No cancer	398 (37.8%)	
1	128 (12.3%)	21 (8.8%)
2	174 (16.7%)	58 (24.3%)
3	142 (13.6%)	60 (25.1%)
4	162 (15.5%)	78 (32.6%)
5	43 (4.1%)	21 (8.8%)

Abbreviations: IQR, interquartile range; ISUP, International Society of Urological Pathology; PI‐RADS, prostate imaging reporting and data system; PSA, prostate‐specific antigen; RP, radical prostatectomy.

Detailed baseline characteristics of the overall cohort and the RP cohort are shown in Table 1.

**Table 2:** Histological biopsy outcomes stratified by PI‐RADS scores of the index lesion.

	TB + SB				TB		SB		TB + PLT 5 mm		TB + PLT 10 mm		TB + PLT 15 mm	
	overall	no PC	iPC	sPC	iPC	sPC	iPC	sPC	iPC	sPC	iPC	sPC	iPC	sPC
overall	1043	394	128	521	85 (64.0%)	423 (81.2%)	138 (107.8%)	483 (92.7%)	108 (84.3%)	471 (90.4%)	117 (91.4%)	495 (95.0%)	134 (104.7%)	503 (96.5%)
PI‐RADS ≤ 2	97	44	11	42	5 (45.5%)	33 (78.6%)	12 (109.1%)	39 (92.9%)	8 (72.7%)	37 (88.1%)	8 (72.7%)	42 (100%)	9 (81.8%)	42 (100%)
PI‐RADS 3	170	105	26	39	12 (46.2%)	19 (48.7%)	26 (100%)	37 (94.9%)	18 (69.2%)	25 (64.1%)	20 (76.9%)	31 (79.5%)	23 (88.5%)	33 (84.6%)
PI‐RADS 4	530	205	84	241	61 (72.6%)	179 (74.3%)	88 (104.8%)	222 (92.1%)	74 (88.1%)	213 (88.4%)	81 (96.4%)	224 (92.9%)	93 (110.7%)	230 (95.4%)
PI‐RADS 5	246	40	7	200	7 (100%)	192 (96.0%)	12 (171.4%)	185 (92.5%)	8 (87.5%)	196 (98.0%)	8 (87.5%)	198 (99.0%)	9 (128.6%)	198 (99.0%)

Abbreviations: iPC, insignificant PC; PC, prostate cancer; PI‐RADS, prostate imaging reporting and data system; PLT, perlesional template; SB, systematic biopsy; sPC, significant PC; TB, targeted biopsy.

### 3.2 Spatial Distribution of Prostate Cancer Missed by MRI‐Targeted Biopsy

**Figure 3:**
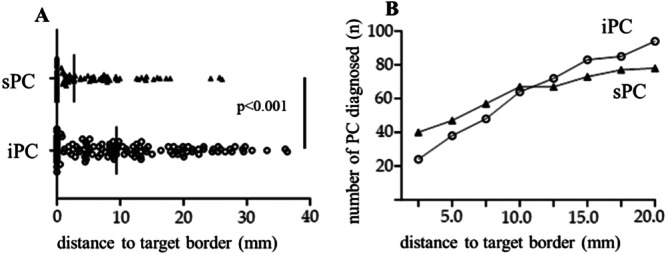
(A) Distance of nearest cancer core to target border stratified for significant versus insignificant prostate cancer. (B) incidence of prostate cancer diagnosed within a certain radius of tumor‐free biopsied MRI target, stratified by significant versus insignificant prostate cancer. The intersection of the lines is approximately at a radius of 10 mm. iPC, insignificant PC; MRI, magnetic resonance imaging; PC, prostate cancer; sPC, significant PC.

Significant PC was identified solely by SB in 98 patients. In our cohort, 55/98 cases (56%) were classified as targeting errors, defined as detection of sPC in an SB core located within the same sextant as a cancer‐negative MRI‐targeted lesion. In 37/98 patients, sPC was detected in an adjacent sextant. In 6/98 patients, sPC was located two or more sextants away from the MRI lesion. Overall, twelve prostate cancers with ISUP ≥ 4 were missed by TB. In these cases, the nearest cancer‐positive SB core was located in the same or an adjacent sextant (ISUP 4: 6/11 same sextant, 5/11 adjacent sextant; ISUP 5: 1/1 same sextant). The mean distance from a cancer‐free MRI lesion to the nearest iPC core was 12.4 mm (SD 9.8). The corresponding mean distance to the nearest sPC core was 7.8 mm (SD 9.0), which was significantly shorter (*p* < 0.001). Figure 3 illustrates cumulative cancer detection within increasing radial distances from the lesion border, stratified by sPC and iPC. With increasing distance from the lesion border, the cumulative number of additionally detected sPC cases increased initially and reached a plateau at approximately 10 mm, whereas the cumulative number of additionally detected iPC cases continued to increase in an approximately linear manner. The frequency curves intersect at approximately 10 mm.

### 3.3 Effects of a Perilesional Template

**Figure 4:**
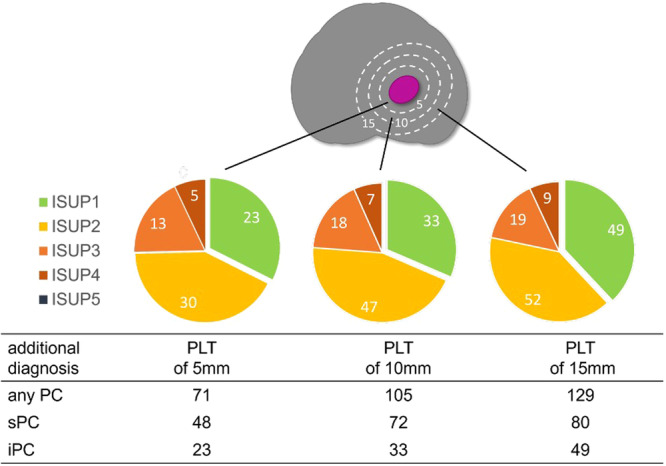
Additionally diagnosed PC with perilesional template (5 mm, 10 mm,15 mm), stratified by ISUP grade. iPC, insignificant PC; ISUP, International Society of Urological Pathology; PLT, perlesional template; PC: prostate cancer; sPC, significant PC. [Color figure can be viewed at wileyonlinelibrary.com]

Using combined biopsy as the reference standard (100%), the hypothetical patient‐based overall cancer detection rate was 89.2%, 94.3%, and 98.2% for 5‐, 10‐, and 15 mm PLTs, respectively, compared with 78.3% for TB alone. Relative to TB alone, this corresponded to 48, 72, and 80 additional diagnoses of sPC for the 5‐, 10‐, and 15 mm PLTs, respectively. A detailed ISUP‐stratified analysis is shown in Figure 4.

Using a 10 mm PLT without SB, 26/1043 (2.5%) sPC cases would have been missed; 15/26 of these were ISUP ≥ 3. Conversely, restricting sampling to a 10 mm PLT would have avoided 39/1043 (3.7%) iPC diagnoses.

Using the combined biopsy as the reference standard (100%), the proportion of sPC that would have been detected using hypothetical 5, 10, and 15 mm PLTs was 88.4%, 92.9%, and 95.4% for PI‐RADS 4 lesions and 98%, 99%, and 99% for PI‐RADS 5 lesions, respectively. Detailed histological biopsy outcomes stratified by PI‐RADS scores of the index lesions are shown in Table 2.

### 3.4 Upgrading at Radical Prostatectomy According to Biopsy Strategy

**Figure 5:**
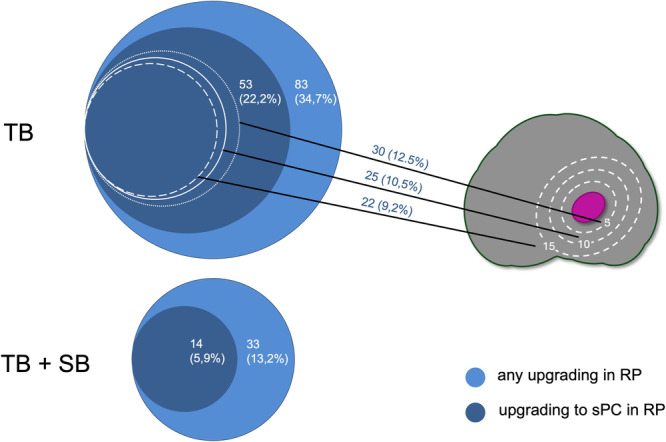
Upgrading of Cancer Grade Group in whole‐mount histopathological analysis, according to biopsy method in 239 patients with radical prostatectomy. Upgrading to sPC at RP decreased from 22.2% with TB alone to 12.5%, 10.5%, and 9.2% with the addition of 5, 10, and 15 mm perilesional templates, respectively. RP: radical prostatectomy; SB: systematic biopsy; sPC: significant prostate cancer; TB: targeted biopsy. [Color figure can be viewed at wileyonlinelibrary.com]

Among the 239 men who underwent RP at our institution, 33/239 (13.2%) experienced upgrading of the ISUP grade group on whole‐mount histopathology, including 14/239 (5.9%) upgrades to sPC. Based on TB alone, upgrading would have occurred in 83/239 (34.7%) cases, with 53/239 (22.2%) upgrades to sPC. The use of PLTs of 5, 10, and 15 mm would have reduced the number of upgrades to sPC to 30/239 (12.5%), 25/239 (10.5%), and 22/239 (9.2%), respectively. Detailed results are shown in Figure 5.

## 4 Discussion

Since TB alone continues to miss a relevant proportion of sPC, SB remains recommended in PC diagnostics. Based on the hypothesis that the spatial distribution of sPC and iPC detected by SB differs in relation to MRI lesions with negative TB findings, our data support targeting error as a major contributor to missed sPC. Against this background, the present study evaluated the effect of a PLT on the detection rates of sPC and iPC compared with the combined biopsy, while potentially reducing procedural invasiveness.

In our cohort of 1043 men, discordant TB and SB results were observed in 172 patients, indicating a clinically relevant diagnostic impact if SB were omitted. Spatial analysis showed that most sPC cores were located close to the MRI target lesion, whereas iPC cores were detected at significantly greater distances, supporting the hypothesis that targeting error is a major contributor to missed sPC.

In a cohort of 2103 biopsies, Williams et al. identified 41 cases in which TB failed to detect sPC and attributed 51.2% of these misses to targeting error [4].

We found that with increasing distance from the lesion border, cumulative detection of sPC increased up to approximately 10 mm and plateaued thereafter, whereas cumulative detection of iPC continued to increase linearly. The intersection of the curves at around 10 mm indicates a spatial threshold at which most sPC is captured. Importantly, these findings provide a data‐driven rationale for the 10 mm perilesional margin currently recommended in guidelines, which has thus far been largely arbitrarily defined. Expanding the margin to 15 mm did not meaningfully increase sPC detection but increased iPC detection, supporting the 10 mm threshold.

Comparable spatial distributions have been reported by Brisbane et al., who showed that 90% of sPC was located within a 10 mm perilesional margin and 99% within a 20 mm margin [7]. Ramen et al. confirmed in their retrospective analysis that 98% of sPC cores were located within 20 mm of the MRI target, whereas ISUP 1 and tumor‐free cores were more widely distributed [10]. Together, these findings support the spatial concentration of sPC around MRI lesions and the rationale for a PLT.

Several retrospective analyses have evaluated PLT‐based approaches. Hagens et al. found in their analysis of 235 cases that TB + PLT detected 96.8% of sPC while avoiding iPC in 4.7% [3, 4]. Tschertadwan et al. demonstrated detection of 99% of sPC using TB plus target‐saturation biopsy [11]. In both studies, however, the PLT was sector‐based rather than defined by a fixed radial margin.

Studies defining a strict 10 mm PLT reported sPC detection rates of 86%, 90%, and 87.5%, while reducing iPC diagnoses by 5.3%, 10.1%, and 1.5%, respectively [7, 12, 13].

In subgroup analyses according to PI‐RADS category, we observed differential effects of the PLT. In PI‐RADS 5 lesions, most sPC was already detected within the MRI‐defined target, limiting additional benefit from PLT. In PI‐RADS 4 lesions, expanding the perilesional margin increased sPC detection, possibly reflecting greater susceptibility to targeting imprecision. In PI‐RADS 3 lesions, no clear plateau was observed, suggesting mechanisms beyond targeting error.

Consistently, the prospective MIRAGE study [14] showed that adding perilesional biopsies to TB improved GG ≥ 2 detection, particularly in biopsy‐naïve men and PI‐RADS 4–5 lesions, while distant sampling added little value. Notably, the geometric extent of the perilesional zone was not defined, whereas our analysis provides a data‐driven 10 mm threshold. This aligns with findings by Lahoud et al., who reported no significant benefit of perilesional sampling in PI‐RADS 3 lesions [15]. This raises the question of whether biopsy strategy should be individualized based on patient risk and MRI lesion characteristics.

Prospective data remain limited. Lombardo et al. performed transrectal fusion biopsy in 262 patients, adding three biopsies from a 10 mm PLT to SB and TB; 80% of sPC were detected by TB and PLT cores [16]. Novara et al. observed a 70% sPC detection rate using TB plus four perilesional cores [17]. Duwe et al. also reported lower sPC detection with TB plus perilesional biopsy compared with combined biopsy [18]. Reported sPC detection rates of 70%–80% indicate that PLT cannot yet replace SB. Differences may relate to the limited sample size and number of perilesional cores. The introduction of an adjusted biopsy strategy combining TB with a PLT only may reduce the total number of biopsy cores and potentially decrease patient burden and overdiagnosis.

However, modification of established diagnostic pathways must be balanced against the risk of missing sPC. While several studies, including the present analysis, report sPC detection rates exceeding 90% with TB plus PLT, other prospective data demonstrate lower detection rates. Therefore, replacement of SB with PLT cannot currently be recommended.

Taken together, our findings provide a rationale for the prospective evaluation of a perilesional biopsy strategy as a potential refinement of current diagnostic pathways. A prospective pilot study investigating a combined TB and 10 mm PLT approach has been initiated, with preliminary results presented in abstract form (DRKS00031670) [19]. Further multicenter prospective validation is required to robustly assess diagnostic performance, safety, and clinical impact before broader implementation.

## 5 Strengths and Limitations

This study provides a comprehensive evaluation of a large pFBx cohort from a high‐volume center. Strengths include the standardized biopsy technique and detailed spatial analysis of biopsy cores in relation to MRI target lesions. Several limitations should be acknowledged. First, the retrospective design inherently limits causal inference. Therefore, this analysis should be considered primarily hypothesis‐generating rather than intended to provide direct guidance for clinical practice. Prospective analyses have to follow. Second, detailed quality metrics of MRI acquisition, interpretation, and PI‐RADS lesion grading were not available, particularly in discordant cases. Third, men with prior biopsies, including biopsy‐negative patients and those under active surveillance, were included, increasing heterogeneity but reflecting real‐world clinical practice. Finally, all biopsies were performed using a robotic transperineal platform. Therefore, the generalizability of our findings to other biopsy systems or the transrectal approach remains uncertain.

## 6 Conclusion

Our results demonstrate that missed sPC is predominantly located near to MRI target lesions, whereas iPC is more widely distributed. A PLT with a radius of approximately 10 mm captures the majority of sPC while limiting additional detection of iPC, supporting the use of PLT as a spatially rational alternative to SB in selected settings. Prospective validation is required before clinical implementation.

## Funding

The authors have nothing to report.

## Ethics Statement

This single‐center retrospective study was approved by the local Ethics Committee (Lead Investigating Center, UHF: ETK 21‐1191, 07 April 2021) and conducted in accordance with the Declaration of Helsinki.

## Conflicts of Interest

The authors declare no conflicts of interest.

## Data Availability

The data that support the findings of this study are available from the corresponding author upon reasonable request. Due to institutional and data protection regulations, the data are not publicly available.
